# Regulation of Amino Acid Transporters by Cell Surface Receptors

**DOI:** 10.3390/antiox15050619

**Published:** 2026-05-14

**Authors:** Chiara Brignola, Myrhiam Cassese, Stefano Marrone, Teresa Esposito, Vincenza Barresi, Gabriella Esposito, Daniele Filippo Condorelli, Rosario Ammendola, Fabio Cattaneo

**Affiliations:** 1 Department of Molecular Medicine and Medical Biotechnology, University of Naples Federico II, Via S. Pansini 5, 80131 Naples, Italy; chiara.brignola@unina.it (C.B.); myrhiam.cassese@unina.it (M.C.); stefan.marrone@studenti.unina.it (S.M.); teresa.esposito28@studenti.unina.it (T.E.); gabriella.esposito@unina.it (G.E.); rosario.ammendola@unina.it (R.A.); 2 Department of Biomedical and Biotechnological Sciences, University of Catania, Via Santa Sofia 97, 95123 Catania, Italy; vincenza.barresi@unict.it (V.B.); daniele.condorelli@unict.it (D.F.C.)

**Keywords:** solute carrier proteins, amino acid transporters, G protein-coupled receptors, receptor tyrosine kinases, NADPH oxidase, reactive oxygen species

## Abstract

Cancer progression is closely linked to the enhanced uptake of extracellular amino acids, mediated by specific transporters that support biosynthesis, metabolic activity, and energy production through the tricarboxylic acid cycle. By increasing the expression of these transporters, tumor cells secure a continuous amino acid supply that sustains the proliferation, metabolic balance, and activation of major signaling pathways. While most studies have emphasized post-translational control of amino acid transporters, such as phosphorylation, ubiquitination, glycosylation, and palmitoylation, emerging evidence highlights regulatory crosstalk between these transporters and other membrane proteins, including G protein-coupled receptors and receptor tyrosine kinases. This review summarizes the current literature on the receptor-mediated mechanisms governing amino acid uptake and explores how interactions among families of membrane proteins contribute to the regulation of transporter activity.

## 1. Introduction

Membrane proteins orchestrate communication between the extracellular milieu and the intracellular space through highly regulated signaling and transport mechanisms. Among these, G protein-coupled receptors (GPCRs) represent the largest family of human membrane proteins and constitute a major class of therapeutic targets. Receptor tyrosine kinases (RTKs) are likewise clinically significant transmembrane receptors, particularly in the context of cancer biology, where aberrant RTK signaling drives tumor progression. Solute carrier (SLC) proteins form a third essential group of membrane proteins that maintain cellular homeostasis by mediating the transport of ions, nutrients, and other small molecules.

Extensive evidence supports GPCR-dependent transactivation of RTKs in both physiological and pathological settings. Well-characterized examples include the interaction between G protein-coupled receptor 30 (GPR30) and the epidermal growth factor receptor (EGFR) in breast cancer [1], the functional crosstalk between formyl peptide receptor 1 (FPR1) and TrkA in neural cells [2], FPR1-mediated transactivation of VEGFR2 in endothelial cells [3], FPR2-driven activation of EGFR [4], FPR2-induced c-MET activation in prostate epithelial cells [5], and FPR2 interactions with IGFRβ/IRβ in various lung cancer cell types [6]. Conversely, tyrosine kinase signaling can also feed into GPCR-dependent pathways: cancer cells are capable of transmitting RTK-derived tyrosine-based signals directly to heterotrimeric G proteins, thereby initiating canonical GPCR signaling cascades independently of ligand engagement [7].

Although SLC transporters do not independently trigger oncogenic signaling, their functional interplay with RTKs and GPCRs can profoundly influence cancer cell metabolism. By modulating transporter stability, localization, or activity, these receptor-driven interactions reshape nutrient uptake and metabolic pathway usage, ultimately contributing to cancer metabolic reprogramming. As a result, cooperative or compensatory crosstalk among GPCRs, RTKs, and SLC transporters can significantly modulate SLC-mediated processes, representing a further level of regulatory complexity that has so far been underestimated in molecular oncology.

Among the various forms of metabolic rewiring observed in cancer, the reprogramming of amino acid (AA) metabolism has emerged as a fundamental hallmark of tumorigenesis. AAs not only function as building blocks for protein and nucleic acid synthesis, but also as anaplerotic substrates fueling the tricarboxylic acid (TCA) cycle, contributors to ammonia detoxification, and critical regulators of cellular redox homeostasis. Beyond their metabolic roles, AAs modulate epigenetic programs and activate key signaling pathways that drive tumor growth, invasion, and metastasis [8].

To sustain these demands, cancer cells frequently upregulate multiple solute carrier (SLC) AA transporters, a phenomenon associated with enhanced tumor progression, poor clinical prognosis, and therapeutic resistance. Inhibition of SLC function reduces AA availability, sensitizes tumor cells to conventional anticancer treatments, and ultimately promotes cell death [9].

The SLC superfamily comprises 65 subfamilies and 458 transporters responsible for mediating the transport of amino acids, metal ions, organic anions, sugars, vitamins, and lipids across cellular membranes [10,11]. Passive facilitative or secondary active AA transport is primarily conducted by members of the SLC1, SLC3, SLC6, SLC7, SLC36, SLC38, and SLC43 families. Among these, the Na^+^-independent L-type AA transport (LAT) systems, including LAT1 (SLC7A5), LAT2 (SLC7A8), LAT3 (SLC43A1), and LAT4 (SLC43A2), mediate the uptake of branched chain amino acids (BCAAs), aromatic AAs, large neutral AAs (LNAAs), and other essential AAs [12]. Although these transporters share several features, they differ markedly in substrate specificity, transport mode, tissue distribution, and regulatory mechanisms [11]. LAT1, in particular, operates as an antiporter, importing leucine, isoleucine, phenylalanine, methionine, histidine, tryptophan, valine, and tyrosine in exchange for intracellular substrates such as glutamine [13]. Of note, the tumor-suppressor proteins of the Rb family, like E2F1 and RBL1 in Chr20 and E2F5 in Chr8, are associated with glutamine metabolism [14,15,16].

SLC substrates may also be shared with ligands recognized by other membrane protein families, such as GPCRs. In these cases, SLC-dependent modulation of extracellular ligand availability can directly influence ligand-driven GPCR signaling. This mechanistic intersection has prompted growing interest in the development of small molecules capable of simultaneously targeting SLCs and GPCRs, which may provide synergistic opportunities for multifunctional cancer therapies [17].

In this review, we investigate the regulation of amino acid uptake mediated by receptor tyrosine kinases (RTKs) and G protein-coupled receptors (GPCRs) and delineate the signaling crosstalk that connects these membrane protein families. By integrating these mechanistic insights, we aim to highlight emerging interfaces between nutrient transport and receptor-driven oncogenic signaling.

## 2. SLCs Regulation by RTKs

### 2.1. Anaplastic Lymphoma Kinase Regulates SLC3A2

Anaplastic lymphoma kinase (ALK) is a member of the RTK family characterized by an extracellular ligand-binding domain, a single-pass transmembrane region, and an intracellular tyrosine kinase domain [18]. The heavy-chain glycoprotein 4F2hc (CD98), encoded by the *SLC3A2* gene on chromosome 1, is a type II transmembrane protein involved in AA trafficking and polyamine transport [19]. 4F2hc forms disulfide linked heterodimers with several light-chain AA transporters, including LAT1, LAT2, SLC7A11/xCT, and SLC7A5, which are essential for their membrane stability, subcellular localization, transport function, and substrate recruitment [20,21,22]. Enhanced expression of SLC3A2/SLC7A5 and SLC3A2/SLC7A11 complexes is associated with malignant transformation, tumor progression, and poor clinical outcome [23,24].

**Figure 1:**
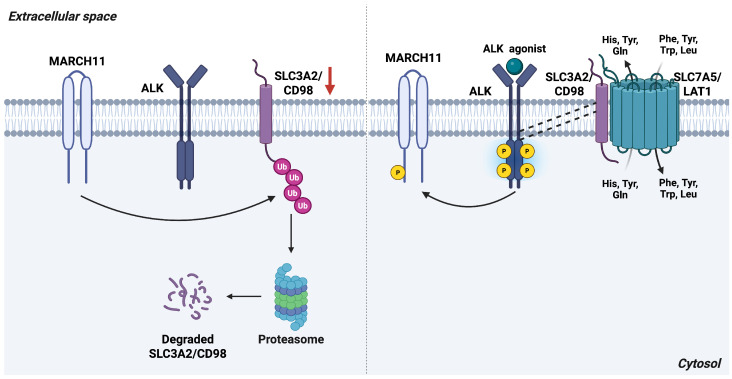
ALK receptor promotes SLC3A2/CD98 stabilization. Left panel: When ALK is inactive, the E3 ubiquitin ligase MARCH11 remains unphosphorylated and active, leading to ubiquitination and proteasomal degradation of SLC3A2/CD98. Right panel: Upon ALK activation, MARCH11 becomes tyrosine-phosphorylated and inactivated, preventing SLC3A2/CD98 degradation. Activated ALK also associates with CD98, enhancing its stability at the plasma membrane and facilitating heterodimer formation with SLC7A5/LAT1, thereby supporting amino acid transport. *Abbreviations*: ALK, anaplastic lymphoma kinase; Gln, glutamine; His, histidine; LAT1, L-type amino acid transporter 1; Leu, leucine; MARCH11, membrane-associated ring finger 11; Phe, phenylalanine; SLC3A2, solute carrier family 3 member 2; SLC7A5, solute carrier family 7 member 5; Trp, tryptophan; Tyr, tyrosine. Created in BioRender. Cattaneo, F. (2026) License https://BioRender.com/6n1kyr8.

In neuroblastoma (NB) cells, co-immunoprecipitation assay shows that ALK interacts with SLC3A2 and regulates its protein stability in a kinase-dependent manner [25]. Ligand-mediated activation of ALK rapidly increases SLC3A2 protein abundance, whereas pharmacological ALK inhibition results in substantial loss of SLC3A2 expression. This regulatory effect is mediated through ALK-dependent control of SLC3A2 ubiquitination and degradation: ALK inhibition enhances SLC3A2 ubiquitination, leading to decreased protein stability, reduced expression, and diminished transporter function [25] (Figure 1).

Post-translational modifications such as ubiquitination are well established mechanisms controlling the abundance, localization, and activity of SLC transporters [26]. Members of the MARCH family of E3 ubiquitin ligases are themselves regulated by phosphorylation, and several phosphorylation sites on MARCH11 are responsive to ALK signaling [27]. Of note, the autocrine loop activation can occur in cancer cells expressing both ALK and its ligands, providing a self-sustaining growth signal and driving cancer growth and survival [28]. These observations suggest that ALK may stabilize SLC3A2 by modulating the phosphorylation state, and thus the activity, of MARCH ubiquitin ligases. Consistent with this model, ALK inhibition leads to MARCH11 dephosphorylation, which enhances its ubiquitin ligase activity and promotes SLC3A2 ubiquitination. Together, these findings define a regulatory ALK–MARCH11–SLC3A2 axis that supports neuroblastoma cell growth [25].

### 2.2. EGFR Regulates SLC7A11/xCT

The epidermal growth factor receptor (EGFR) is a member of the ErbB family of RTKs, which consists of four closely related receptors: EGFR (ErbB1), HER2/neu (ErbB2), HER3 (ErbB3), and HER4 (ErbB4).

The type II transmembrane glycoprotein system xc(−) is composed of the light-chain subunit SLC7A11/xCT and the heavy-chain subunit SLC3A2 (CD98/4F2hc). Together, these proteins form a heterodimeric antiporter located at the plasma membrane that mediates the import of cystine in exchange for intracellular glutamate. Following its entry into the cytosol, cystine is rapidly reduced to cysteine through an NADPH-dependent reaction. Increased expression of SLC7A11/xCT at the cell surface enhances cystine uptake, thereby supporting the biosynthesis of reduced glutathione (GSH), a key determinant of redox homeostasis in cancer cells [29,30].

**Figure 2:**
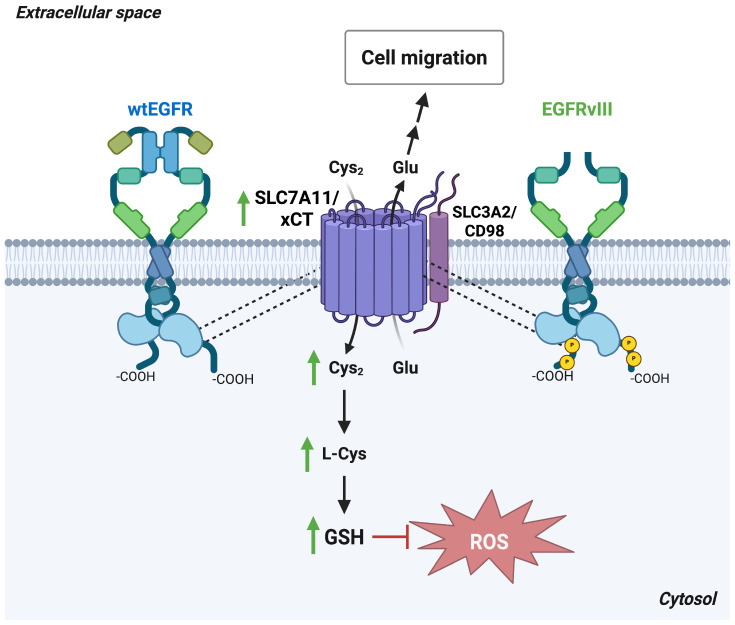
EGFR stabilizes SLC7A11/xCT at the plasma membrane in a kinase-independent manner. In glioma cells, the intracellular domain of both unstimulated wild-type EGFR (wtEGFR) and the constitutively active EGFR variant III (EGFRvIII) mediates binding to the central region of SLC7A11/xCT, thereby stabilizing its expression and membrane localization. This stabilization enhances cystine uptake, increasing intracellular L-cysteine levels and GSH synthesis, which in turn mitigates ROS accumulation. Concurrently, the resulting elevation in glutamate release promotes glioma cell migration and invasion. *Abbreviations*: Cys, cysteine; Cys_2_, cystine; EGFR, epidermal growth factor receptor; Glu, glutamate; GSH, reduced glutathione; ROS, reactive oxygen species; SLC7A11, solute carrier family 7 member 11; xCT, cystine/glutamate antiporter. Created in BioRender. Cattaneo, F. (2026) License https://BioRender.com/6n1kyr8.

In glioma cells, co-immunoprecipitation assay shows that the intracellular domain of EGFR is required for the interaction with SLC7A11/xCT, resulting in increased transporter expression, elevated cystine import, and enhanced GSH production. These effects collectively promote extracellular matrix invasion and bolster the antioxidant capacity of tumor cells. Notably, the constitutively active EGFRvIII mutant, despite lacking the capacity to bind canonical ligands, also associates with SLC7A11/xCT and drives its surface localization. This indicates that the regulation of SLC7A11/xCT by EGFR occurs independently of EGFR kinase activity (Figure 2). Together, these findings highlight the EGFR–SLC7A11/xCT axis as a potential therapeutic target for limiting glioma growth and invasiveness [31].

### 2.3. Discoidin Domain Receptor 1 Regulates SLC7A11

Discoidin domain receptor 1 (DDR1) is a member of the RTK family activated by fibrillar and basement membrane collagens, particularly collagen types I and IV. Upon ligand engagement, DDR1 triggers the downstream signaling pathways that regulate cell proliferation, tumor metabolism, chemotherapeutic response, and overall patient outcomes [32].

**Figure 3:**
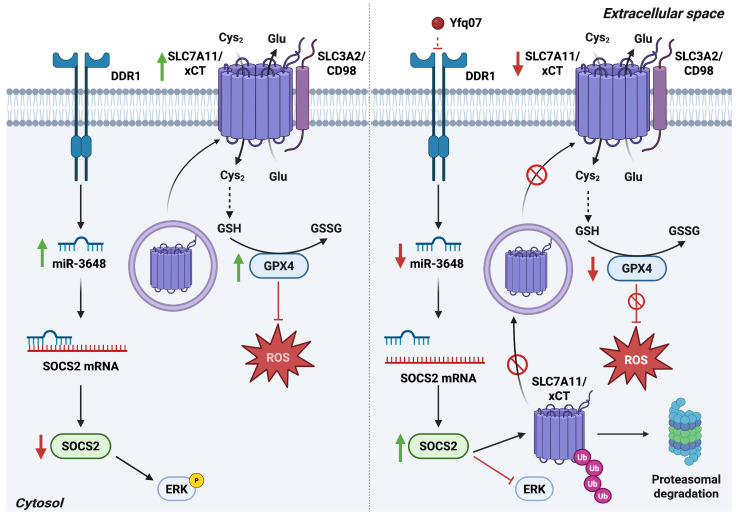
The DDR1–SOCS2 axis regulates SLC7A11/xCT ubiquitination. Left panel: DDR1 activation induces miR-3648 expression, leading to suppression of SOCS2 and enhancement of ERK signaling. Reduced SOCS2 levels increase SLC7A11/xCT and GPX4 protein expression, thereby supporting GSH-dependent antioxidant defenses. Right panel: Pharmacological inhibition of DDR1 by Yfq07 lowers miR-3648 expression, elevates SOCS2 levels, and diminishes ERK signaling. Increased SOCS2 promotes ubiquitination and proteasomal degradation of SLC7A11/xCT, resulting in decreased membrane localization, reduced cystine uptake, and diminished GPX4 levels. Together, these effects elevate ROS accumulation and disrupt redox homeostasis. *Abbreviations*:  Cys_2_, cystine; DDR1, discoidin domain receptor 1; ERK, extracellular signal-regulated kinase; Glu, glutamate; GPX4, glutathione peroxidase 4; GSH, reduced glutathione; GSSG, oxidized glutathione; ROS, reactive oxygen species; SLC3A2, solute carrier family 3 member 2; SLC7A11, solute carrier family 7 member 11; SOCS2, suppressor of cytokine signaling 2; xCT, cystine/glutamate antiporter. Created in BioRender. Cattaneo, F. (2026) License https://BioRender.com/6n1kyr8.

DDR1 is highly expressed in epithelial tissues, and aberrant upregulation has been reported in multiple fibrotic disorders [33], as well as in a wide range of human cancers [34], where it interacts with oncogenic signaling networks to promote tumor progression. Recent findings reveal a regulatory axis linking DDR1 activity to redox balance and ferroptosis sensitivity. Inhibition of DDR1 suppresses the expression of miR 3648, leading to increased levels of the Suppressor of Cytokine Signaling 2 (SOCS2) protein. Elevated SOCS2 attenuates ERK signaling and reduces malignant phenotypes. Moreover, SOCS2 upregulation enhances the ubiquitination and subsequent degradation of the cystine transporter SLC7A11 [35], resulting in diminished cystine import and reduced synthesis of glutathione peroxidase 4 (GPX4), a key enzyme that protects membrane lipids from peroxidation. Consequently, inhibition of DDR1 activates the DDR1–miR 3648–SOCS2–SLC7A11 signaling cascade, leading to increased lipid peroxidation and coordinated downregulation of SLC7A11 and GPX4 at both the mRNA and protein levels [36] (Figure 3).

Ubiquitination typically occurs on lysine residues of substrate proteins, and SLC7A11 contains seven conserved lysine sites that serve as targets for ubiquitin attachment [37]. Once ubiquitinated, SLC7A11 is recognized by the 26S proteasome and degraded, contributing to the induction of ferroptosis. DDR1-dependent upregulation of SOCS2 accelerates this process by promoting SLC7A11 ubiquitination and facilitating ferroptosis in part through STEAP3, the principal ferrireductase responsible for converting Fe^3+^ to Fe^2+^ [35].

Collectively, these findings identify a DDR1-miR 3648-SOCS2-SLC7A11 regulatory axis that integrates collagen-dependent RTK activity with cystine metabolism, redox control, and ferroptotic vulnerability. Targeting this pathway may represent a promising therapeutic strategy to enhance tumor radiosensitivity and improve clinical outcomes.

### 2.4. DDR1 Regulates SLC1A5/ASCT2

SLC1A5 (ASCT2) is a sodium-dependent neutral amino acid transporter localized at the plasma membrane and is broadly expressed across human tissues. The SLC1 transporter family consists of five excitatory amino acid transporters (EAAT1–EAAT5) and two additional members, SLC1A4/ASCT1 and SLC1A5/ASCT2 [38]. Among these transporters, SLC1A5 serves as the primary mediator of glutamine uptake, particularly in tumor cells, where glutamine becomes an essential nutrient supporting biosynthetic and bioenergetic demands. Following import into the cytosol, glutamine can be transported into mitochondria [39], where a mitochondrial splice variant of SLC1A5 facilitates its entry into the mitochondrial matrix [40,41]. Glutamine also participates in transmembrane exchange via SLC7A5/LAT1, exiting the cell in exchange for leucine import, a process that activates the mammalian target of rapamycin complex 1 (mTORC1) signaling and promotes cell growth [42]. Within mitochondria, glutaminases GLS1 and GLS2 convert glutamine to glutamate, which is subsequently processed by glutamate dehydrogenase (GDH) into α ketoglutarate to replenish the TCA cycle. In addition, glutamate serves as a precursor for GSH synthesis, which is essential for maintaining cellular redox homeostasis [43].

Collagen I stimulation enhances DDR1 phosphorylation on tyrosine residues and promotes the stabilization of SLC1A5 by preventing its lysosomal degradation. Notably, DDR1 kinase inhibitors block DDR1 autophosphorylation, but do not impair DDR1-dependent stabilization of SLC1A5, indicating that this regulatory mechanism relies on a kinase-independent interaction between DDR1 and SLC1A5 [34]. Lysosomes play a central role in nutrient-responsive signaling and adaptive metabolic processes [44]. SLC1A5 undergoes extensive glycosylation at the plasma membrane, and such glycosylated membrane proteins are preferentially degraded through lysosomal pathways rather than proteasomal ones.

**Figure 4:**
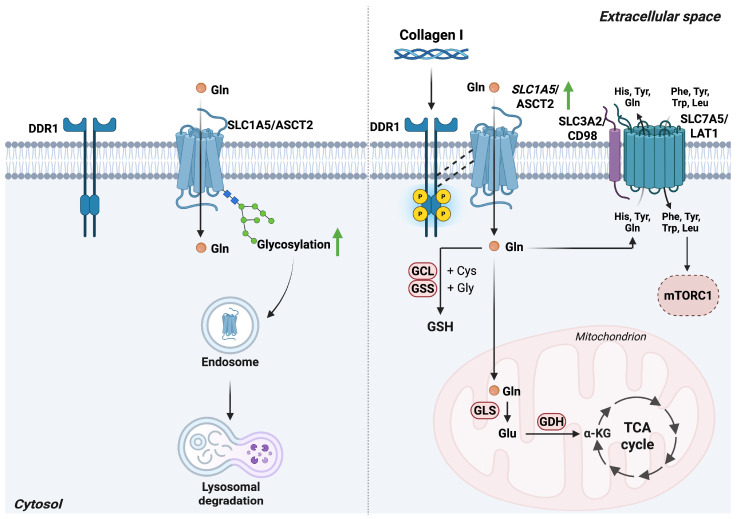
DDR1 signaling enhances SLC1A5/ASCT2 stabilization and drives glutamine-dependent metabolic rewiring. Left panel: In cells lacking DDR1 activity, SLC1A5/ASCT2 undergoes glycosylation that targets it for lysosomal degradation. Right panel: Collagen I-mediated DDR1 activation stabilizes SLC1A5/ASCT2 at the plasma membrane, thereby increasing glutamine uptake. Imported glutamine supports GSH synthesis via sequential GCL and GSS activity and/or fuels the TCA cycle following its conversion to glutamate by GLS and then to α-ketoglutarate by GDH. Additionally, glutamine facilitates amino acid exchange through SLC7A5/LAT1, promoting leucine import and subsequent activation of mTORC1. *Abbreviations*: ASCT2, alanine-serine-cysteine transporter 2; DDR1, discoidin domain receptor 1; GDH, glutamate dehydrogenase; GCL, glutamate-cysteine ligase; Gln, glutamine; GLS, glutaminase; GSS, glutathione synthetase; His, histidine; LAT1, L-type amino acid transporter 1; Leu, leucine; mTORC1, mechanistic target of rapamycin complex 1; Phe, phenylalanine; SLC1A5, solute carrier family 1 member 5; SLC7A5, solute carrier family 7 member 5; TCA, tricarboxylic acid cycle; Trp, tryptophan; Tyr, tyrosine. Created in BioRender. Cattaneo, F. (2026) License https://BioRender.com/6n1kyr8.

Because SLC1A5-mediated glutamine uptake and SLC7A5-mediated leucine import converge to support tumor proliferation, their coordinated regulation is integral to mTORC1 activity (Figure 4).

mTORC1, a master regulator of cell growth, senses intracellular fluctuations in glutamine and leucine levels, and thereby in SLC1A5 and SLC7A5 activity, and adjusts downstream signaling accordingly. Intracellular leucine is detected either by leucyl tRNA synthetase or via the sestrin2–GATOR signaling module, both of which convey leucine availability to the mTORC1 regulatory complex. Thus, DDR1-dependent stabilization of SLC1A5 underscores the importance of glutamine metabolism and its upstream regulatory mechanisms in driving cancer progression [34].

### 2.5. Insulin-like Growth Factor 1 Receptor Regulates SLC7A11/xCT and SLC40A1

The insulin-like growth factor 1 receptor (IGF1R) is a member of the RTK family and is activated upon binding to its cognate ligand, insulin-like growth factor 1 (IGF 1). SLC40A1, also known as Ferroportin 1, is a transmembrane iron exporter and the only known protein capable of mediating cellular iron efflux.

**Figure 5:**
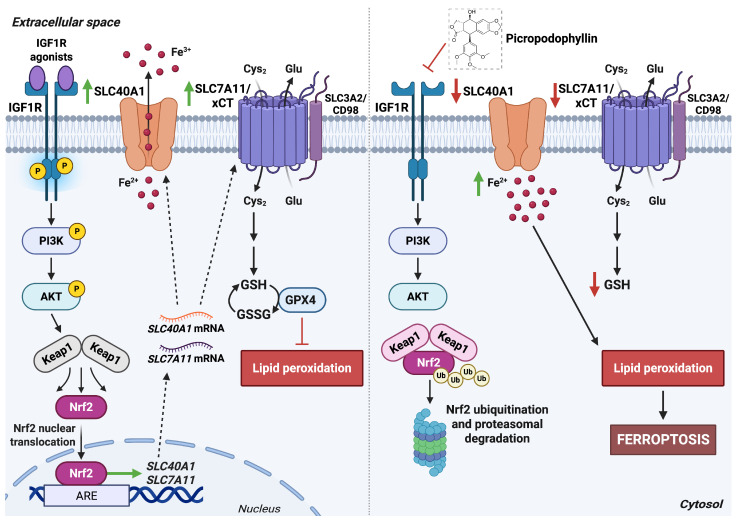
IGF1R activity regulates SLC7A11/xCT and SLC40A1 expression. Left panel: Activation of IGF1R by specific agonists stimulates the PI3K/AKT pathway, promoting Keap1–Nrf2 dissociation and enabling Nrf2 nuclear translocation. Nuclear Nrf2 induces the transcription of target genes such as SLC40A1, with subsequent reduction in intracellular iron pool, and SLC7A11, which enhances cystine uptake for GSH synthesis. Together, these responses limit lipid peroxidation and support redox homeostasis. Right panel: The natural IGF1R inhibitor picropodophyllin suppresses PI3K/AKT signaling, resulting in Nrf2 ubiquitination and proteasomal degradation. Reduced expression of SLC40A1 and SLC7A11/xCT leads to intracellular iron accumulation, impaired GSH biosynthesis, and decreased GPX4 activity, collectively promoting ferroptosis. *Abbreviations*:  Cys_2_, cystine; Glu, glutamate; GPX4, glutathione peroxidase 4; GSH, reduced glutathione; GSSG, oxidized glutathione; IGF1R, insulin-like growth factor 1 receptor; Keap1, Kelch-like ECH-associated protein 1; Nrf2, nuclear factor erythroid 2–related factor 2; PI3K, phosphatidylinositol 3-kinase; SLC40A1, solute carrier family 40 member 1; SLC7A11, solute carrier family 7 member 11; xCT, cystine/glutamate antiporter. Created in BioRender. Cattaneo, F. (2026) License https://BioRender.com/6n1kyr8.

Accumulating evidence links IGF1R signaling to the cellular response to oxidative stress, implicating this pathway in the regulation of lipid peroxidation-driven ferroptosis. A significant correlation has been reported between IGF1R expression and key ferroptosis regulators, including NRF2, SLC7A11, and SLC40A1, suggesting that inhibition of IGF1R may be an effective strategy to induce ferroptosis in cancer cells [45]. High IGF1R levels, or stimulation with IGF1R agonists, activate the IGF1R/PI3K/AKT signaling cascade, resulting in NRF2 upregulation. Within the promoter regions of the SLC7A11 and SLC40A1 genes, two NRF2-responsive binding sites have been identified, supporting a model in which NRF2 transcriptionally enhances the expression of these transporters [45]. Conversely, IGF1R inhibition markedly suppresses PI3K and AKT activation and reduces the expression of NRF2, SLC7A11, and SLC40A1 [45,46]. This suppression alters several ferroptosis-related processes, including increased intracellular Fe^2+^ accumulation, elevated levels of lipid-derived reactive species and malondialdehyde, and reduced GSH levels. Mechanistically, blockade of the IGF1R/PI3K/NRF2 axis downregulates SLC7A11 and SLC40A1, thereby limiting cystine import, impairing GSH synthesis, and promoting Fe^2+^ overload, all of which drive lipid hydroperoxide accumulation and ferroptotic cell death (Figure 5) [47].

Together, these findings identify IGF1R as a key regulator of ferroptosis through coordinated control of redox metabolism and iron homeostasis. Targeting the IGF1R–PI3K–NRF2 signaling axis may therefore provide a promising therapeutic strategy for inducing ferroptosis and overcoming treatment resistance in cancer.

### 2.6. Fibroblast Growth Factor Receptor 1 Regulates SLC1A5

The fibroblast growth factor receptor 1 (FGFR1) is a member of the receptor tyrosine kinase (RTK) superfamily that regulates crucial cellular processes, including proliferation, differentiation, and survival, through interactions with specific fibroblast growth factor ligands [48]. Aberrant FGFR1 expression has been documented across a wide spectrum of solid tumors and hematologic malignancies [49], positioning FGFR1 inhibitors as a promising class of antitumoral agents. Nevertheless, both intrinsic and acquired resistance substantially limit the long-term efficacy of FGFR1-targeted therapies [50].

A central mechanism underlying resistance involves the robust induction of activating transcription factor 4 (ATF4), a key effector of the integrated stress response (ISR) and a major driver of FGFR1 inhibitor resistance in T cell acute lymphoblastic leukemia (T ALL) [49]. ATF4 coordinates adaptive cellular programs that counter metabolic and oxidative stress, including nutrient scarcity and redox imbalance [51]. Upon FGFR1 inhibition, multiple metabolic pathways associated with cell survival, notably those involved in amino acid metabolism, are upregulated. ATF4 is required for these adaptive responses, as ATF4 depletion eliminates the accumulation of survival promoting metabolites following FGFR1 blockade [49]. The increase in ATF4 expression upon FGFR1 inhibitor treatment is driven by enhanced chromatin accessibility coupled with activation of the GCN2 eIF2α translational stress pathway.

**Figure 6:**
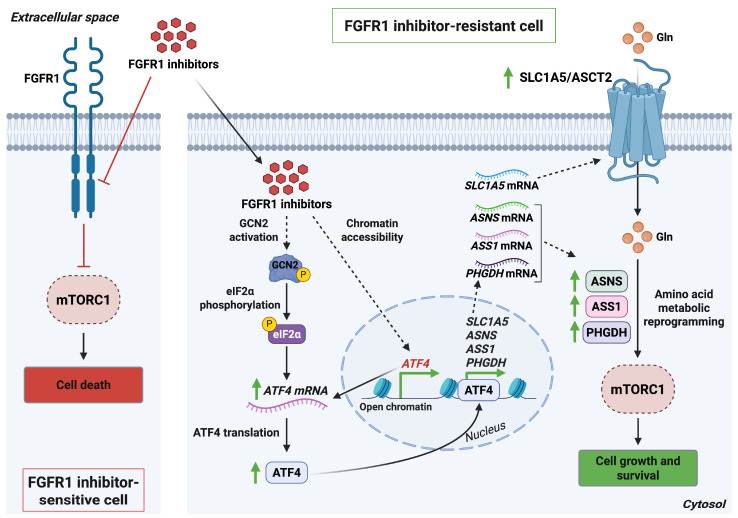
ATF4-driven amino acid uptake via SLC1A5/ASCT2 supports resistance to FGFR1 inhibitors. In FGFR1-inhibitor-sensitive T-ALL cells, FGFR1 blockade suppresses FGFR1 signaling, leading to inhibition of mTORC1 activity and induction of cell death. In contrast, FGFR1-inhibitor-resistant T-ALL cells exhibit increased ATF4 expression upon FGFR1 inhibitor treatment, driven by enhanced chromatin accessibility and activation of the GCN2–eIF2α pathway. ATF4 upregulates a network of metabolic genes, such as *SLC1A5,* *ASNS*, *ASS1*, and *PHGDH*, thereby reprogramming amino acid uptake and metabolism to sustain mTORC1 activation and promote cell survival. *Abbreviations*: ASCT2, alanine-serine-cysteine transporter 2; *ASNS*, asparagine synthetase; *ASS1*, argininosuccinate synthetase 1; ATF4, activating transcription factor 4; eIF2α, eukaryotic initiation factor 2 subunit 1; FGFR1, fibroblast growth factor receptor 1; GCN2, general control non-derepressible 2; Gln, glutamine; mTORC1, mammalian target of rapamycin complex 1; *PHGDH*, phosphoglycerate dehydrogenase; *SLC1A5*, solute carrier family 1 member 5; T-ALL, T-cell acute lymphoblastic leukemia. Created in BioRender. Cattaneo, F. (2026) License https://BioRender.com/6n1kyr8.

Once upregulated, ATF4 promotes metabolic reprogramming through upregulation of the amino acid transporter SLC1A5 and subsequent activation of mTORC1, both of which contribute to therapeutic resistance in T ALL [49] (Figure 6). Consistently, silencing SLC1A5 suppresses mTORC1 signaling and markedly sensitizes cells to FGFR1 inhibition [52]. Conversely, elevated levels of arginine, asparagine, and other essential amino acids can potentiate mTORC1 activation [53]. Collectively, these findings identify ATF4-dependent amino acid uptake via SLC1A5 as a pivotal driver of mTORC1 activation and resistance to FGFR1-targeted therapies, highlighting SLC1A5 inhibition as a compelling therapeutic strategy to overcome FGFR1 inhibitor resistance.

### 2.7. Ephrin Type-A Receptor 2 Regulates SLC1A5

Ephrin type A receptor 2 (EphA2) is a member of the ephrin receptor subfamily within the RTK family. Structurally, EphA2 contains a single intracellular kinase domain and an extracellular region composed of a cysteine-rich domain followed by two fibronectin type III repeats. Its ligands fall into two subclasses, ephrin A and ephrin B, which differ in molecular architecture and membrane anchorage.

EphA2 can transduce signals through two mechanistically distinct modes. In its canonical ligand-dependent configuration, EphA2 mediates forward signaling in normal tissues, where interactions with membrane-bound ephrin ligands suppress cell proliferation and limit invasive behavior. In contrast, when ligand availability is reduced or when EphA2 is transactivated by other RTKs, such as EGFR or HER2, the receptor shifts to a ligand-independent signaling mode that promotes oncogenic processes. Consistent with this paradigm, EphA2 is frequently overexpressed in malignant breast tumors, whereas its ephrin ligands are commonly downregulated, creating a signaling imbalance associated with poor clinical outcomes and decreased responsiveness to anticancer therapies [54].

**Figure 7:**
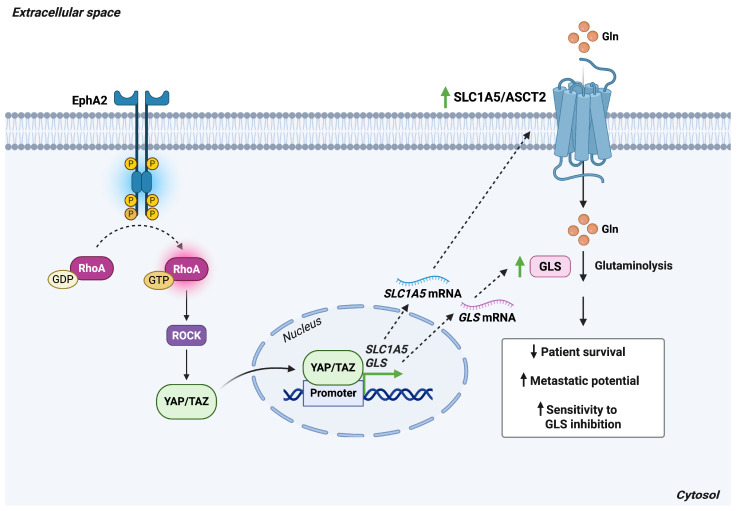
EphA2 signaling induces SLC1A5/ASCT2 expression and enhances glutaminolysis. In HER2-positive breast cancer cells, ligand-independent EphA2 signaling activates the RhoA/ROCK–YAP/TAZ pathway, leading to increased transcription of *SLC1A5* and *GLS*. Elevated expression of these genes promotes glutaminolysis, a metabolic adaptation associated with poor patient survival. Enhanced glutamine metabolism also confers sensitivity to GLS inhibition and supports metastatic progression. *Abbreviations*: ASCT2, alanine-serine-cysteine transporter 2; EphA2, erythropoietin-producing hepatocellular receptor A2; Gln, glutamine; *GLS*, glutaminase; ROCK, Rho-associated protein kinase; *SLC1A5*, solute carrier family 1 member 5; TAZ, WW domain-containing transcription regulator 1; YAP, Yes-associated protein. Created in BioRender. Cattaneo, F. (2026) License https://BioRender.com/6n1kyr8.

Beyond its role in tumor cell signaling, EphA2 has been implicated in the metabolic reprogramming of breast cancer cells. EphA2 overexpression enhances glutamine metabolism [55], a process driven by the nuclear accumulation and activation of the transcriptional coactivator Yes-associated protein (YAP). The activated YAP interacts with transcriptional partners, including members of the TEAD family [54], to modulate the expression of metabolic genes such as those encoding SLC1A5 and glutaminase (GLS) [55]. Elevated EphA2–YAP signaling correlates with reduced patient survival, increased metastatic potential, and heightened sensitivity to glutaminase inhibition (Figure 7). Together, these findings identify the EphA2/YAP/SLC1A5 axis as a previously unrecognized regulatory pathway controlling glutamine metabolism in breast cancer.

## 3. SLCs Regulation by GPCRs

### 3.1. The Super-Conserved Receptors Expressed in the Brain Regulate SLC3A2

The super conserved receptors expressed in the brain (SREBs) constitute a subfamily of orphan GPCRs characterized by a predominant expression in the central nervous system (CNS). This group comprises three highly evolutionarily conserved members, namely SREB1 (GPR27), SREB2 (GPR85), and SREB3 (GPR173) [56,57]. Although SREBs display some sequence similarity to prostaglandin, purinergic, and amine receptor families, and are therefore classified within the rhodopsin like (class A) GPCR superfamily [57,58], their primary sequences lack, or diverge substantially, from many canonical molecular motifs of class A receptors [59]. These atypical structural features have contributed to persisting uncertainties regarding their physiological roles.

A defining feature of the SREB family is the presence of a highly conserved amino acid motif within the third intracellular loop, suggesting the existence of shared protein interaction partners. One such interactor is SLC3A2 (4F2hc, CD98), which heterodimerizes with members of the SLC7 light-chain family to form system L-amino acid transporters. These heterodimers mediate the cellular uptake of large neutral amino acids, including leucine, phenylalanine, and tryptophan [56,60].

**Figure 8:**
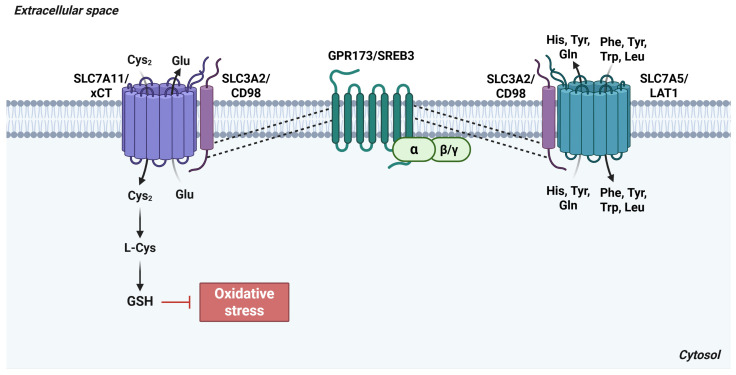
Interaction between GPR173/SREB3 and SLC3A2/CD98. In the central nervous system, GPR173/SREB3 associates with SLC3A2/CD98, a heavy-chain transmembrane protein that partners with SLC7-family light-chain transporters. These include SLC7A5/LAT1, which supports the import of large neutral amino acids, and SLC7A11/xCT, which contributes to redox homeostasis by mediating cystine uptake. *Abbreviations*:  L-Cys, cysteine; Cys_2_, cystine; Glu, glutamate; Gln, glutamine; GPR173, probable G-protein coupled receptor 173; GSH, reduced glutathione; His, histidine; LAT1, L-type amino acid transporter 1; Leu, leucine; Phe, phenylalanine; SLC3A2, solute carrier family 3 member 2; SLC7A5, solute carrier family 7 member 5; SLC7A11, solute carrier family 7 member 11; SREB3, super conserved receptor expressed in brain 3; Trp, tryptophan; Tyr, tyrosine; xCT, cystine/glutamate antiporter. Created in BioRender. Cattaneo, F. (2026) License https://BioRender.com/6n1kyr8.

Within the CNS, the SLC3A2/SLC7A11 heterodimer functions as the core component of the system xc^−^ antiporter, the principal cystine/glutamate exchanger in neurons and glial cells. This transporter regulates cystine import and glutamate efflux and plays a critical role in maintaining intracellular redox homeostasis and protecting against oxidative stress (Figure 8) [61]. Thus, through their association with SLC3A2, which pairs with SLC7A11 to form system xc^−^, SREBs may modulate amino acid transport dynamics and redox regulatory pathways in the CNS.

### 3.2. Taste Receptors Type 1 Member 1 and 3 Regulate SLC3A2 and SLC1A5

The GPCR complex, composed by taste receptor type 1 members 1 and 3 (T1R1/T1R3), functions as an amino acid sensor initially identified in gustatory neurons. By binding L amino acids, mainly L glutamine, it mediates the detection of umami taste [62]. A structurally related receptor complex, T1R2/T1R3, is also expressed in gustatory cells and serves as the principal sweet taste receptor. Beyond its role in taste perception, T1R2/T1R3 operates as a glucose sensor in both the intestinal epithelium and the hypothalamus, thereby contributing to nutrient sensing and metabolic regulation [63,64].

T1R1/T1R3 is a key component of cellular amino acid-sensing mechanisms, providing an early extracellular cue of amino acid availability that informs intracellular energy-sensing pathways, including mTORC1 [65]. Genetic depletion of T1R1/T1R3 triggers a cellular response characteristic of amino acid scarcity: cells upregulate SLC1A5, the primary transporter of glutamine, and increase SLC3A2 mRNA levels. SLC3A2 forms disulfide linked heterodimers with several SLC7 family light-chain transporters, thereby supporting enhanced uptake of amino acids and monocarboxylates. These compensatory transcriptional changes strongly support the role of T1R1/T1R3 as an essential extracellular amino acid sensor. Moreover, T1R1/T1R3 depletion leads to downregulation of two negative regulators of mTORC1 (REDD1 and TSC2), reflecting an attempt to counterbalance impaired receptor-mediated signaling and restore mTORC1 activation.

**Figure 9:**
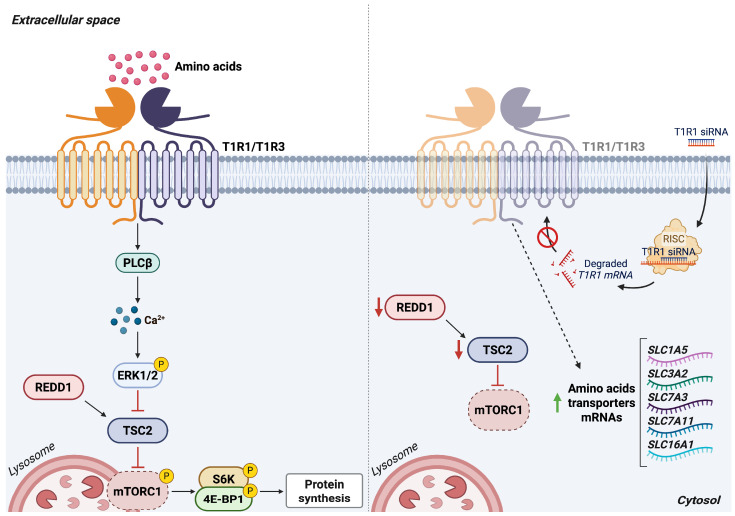
Silencing the amino acid sensor T1R1/T1R3 triggers compensatory upregulation of SLC transporters. Left panel: Amino acid-mediated activation of T1R1/T1R3 stimulates PLCβ, leading to intracellular Ca^2+^ release and ERK1/2 phosphorylation. Downregulation of the mTORC1-negative regulators TSC2 and REDD1 promotes mTORC1 recruitment to lysosomes and phosphorylation of its downstream effectors S6K and 4E-BP1, thereby enhancing protein synthesis. Right panel: Silencing T1R1/T1R3 disrupts PLCβ–ERK signaling, impairing proper mTORC1 localization and activation. This triggers a compensatory response characterized by reduced TSC2 and REDD1 levels and increased amino acid uptake through transcriptional upregulation of multiple SLC transporters. *Abbreviations*: 4E-BP1, eukaryotic initiation factor 4E-binding protein 1; ERK1/2, extracellular signal-regulated kinases 1/2; mTORC1, mammalian target of rapamycin complex 1; PLCβ, phospholipase C-β; REDD1, regulated in development and DNA damage-response 1; S6K, ribosomal protein S6 kinase; SLC1A5, solute carrier family 1 member 5; SLC16A1, solute carrier family 16 member 1; SLC3A2, solute carrier family 3 member 2; SLC7A3, solute carrier family 7 member 3; SLC7A11, solute carrier family 7 member 11; T1R1/3, taste receptors type 1 member 1/3; TSC2, tuberous sclerosis complex 2. Created in BioRender. Cattaneo, F. (2026) License https://BioRender.com/6n1kyr8.

Collectively, these findings demonstrate that T1R1/T1R3 is required for coordinating SLC1A5- and SLC3A2-dependent amino acid trafficking and for maintaining appropriate mTORC1 signaling (Figure 9). Owing to its accessibility to pharmacological modulation, this GPCR complex represents a promising therapeutic target for several human diseases.

### 3.3. Formyl-Peptide Receptor 2 Regulates SLC7A11/xCT

Formyl peptide receptors 1, 2, and 3 (FPR1, FPR2, and FPR3) are members of the GPCR superfamily and constitute a subgroup of receptors coupled to inhibitory G proteins [66]. These receptors respond to specific agonists and activate distinct signaling pathways that regulate cell adhesion, chemotaxis, and NADPH oxidase (NOX)-dependent superoxide production across multiple cell types [67,68]. FPR2 is localized on both the plasma membrane and nuclear membranes of various cell types [66,69]. It mediates chemotactic signals and exhibits context-dependent functions, promoting either pro-inflammatory or anti-inflammatory responses depending on the nature of the ligand and/or interactions with other FPR isoforms [70]. In addition, FPR2 induces the transactivation of several RTKs, thereby initiating multiple intracellular signaling pathways [4,5,6].

The ligand repertoire for FPR2 includes the synthetic peptide WKYMVm [5,71], the anti-inflammatory mediator annexin A1 (ANXA1) [72,73], and lipoxin A4 (LXA4), which exerts both anti-inflammatory and pro-resolving effects [72]. Downstream events triggered by FPR2 activation encompass the stimulation of multiple kinases [74,75], phosphorylation of signaling and non-signaling proteins [76], p47phox phosphorylation and NOX activation [77], as well as NOX-dependent activation of cPLA2 and 5 LOX [78].

The NOX family comprises seven isoforms (NOX1–5, DUOX1, and DUOX2), which are membrane-associated enzymes whose primary function is reactive oxygen species (ROS) production [79]. Among these, NOX2 is the most broadly expressed isoform in humans [79]. Its activation requires phosphorylation of the cytosolic subunit p47phox and its recruitment, together with other cytosolic components, to the membrane [79]. Once assembled, the active NOX2 complex catalyzes the transfer of electrons from NADPH to molecular oxygen, generating superoxide anion (O_2_^•−^) [80]. While high ROS concentrations promote cell death, lower ROS levels play essential physiological roles in signaling and in maintaining cellular homeostasis [81].

Tumor cells undergo profound metabolic reprogramming, which includes a marked increase in reactive oxygen species (ROS) production [82,83]. To counteract ROS elevation and maintain redox balance, eukaryotic cells rely on a variety of antioxidant defense mechanisms. During GSH synthesis, excess intracellular cysteine is exported and oxidized to cystine, which is then continuously imported via the SLC7A11/xCT antiporter. This transport cycle establishes a cystine–cysteine redox shuttle across the plasma membrane, generating a reduced extracellular environment that supports cancer cell proliferation and survival [84].

In cancer cells, the FPR2 agonists WKYMVm and ANXA1 induce a time-dependent upregulation of SLC7A11/xCT. This regulatory effect requires functional NADPH oxidase activity, as NOX inhibitors prevent the FPR2-dependent increase in SLC7A11/xCT expression [85]. Once transported into the cell, extracellular cystine is reduced to cysteine and used for GSH biosynthesis, thereby contributing to ROS detoxification [85].

Lipid peroxidation is strictly dependent on intracellular GSH levels. Therefore, SLC7A11/xCT inhibition markedly enhances lipid peroxidation, while its overexpression in cancer cells increases GSH production [86]. Because FPR2 activation promotes SLC7A11/xCT upregulation and elevates GSH concentrations, FPR2 stimulation likewise mitigates lipid peroxidation [85].

**Figure 10:**
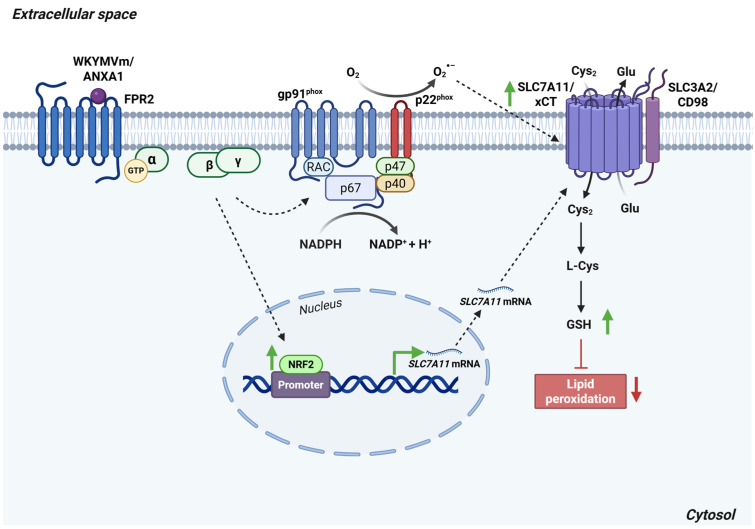
FPR2 regulates SLC7A11/xCT expression and lipid peroxidation. Activation of FPR2 by its endogenous ligand ANXA1 or the synthetic agonist WKYMVm enhances NRF2 nuclear translocation through a NOX-dependent mechanism. Elevated NRF2 activity drives the upregulation of SLC7A11/xCT, which exchanges intracellular glutamate for extracellular cystine. Imported cystine is subsequently reduced to cysteine, the rate-limiting precursor for GSH synthesis. As a result, FPR2 stimulation increases GSH production and mitigates lipid peroxidation. *Abbreviations*: Cys, cysteine; Cys_2_, cystine; FPR2, formyl peptide receptor 2; Glu, glutamate; GSH, reduced glutathione; NRF2, nuclear factor erythroid 2-related factor 2; RAC, Ras-related C3 botulinum toxin substrate 1; SLC3A2, solute carrier family 3 member 2; SLC7A11, solute carrier family 7 member 11; xCT, cystine/glutamate antiporter. Created in BioRender. Cattaneo, F. (2026) License https://BioRender.com/6n1kyr8.

The transcription factors NRF2 and ATF4 are key regulators of SLC7A11/xCT expression [87,88]. NRF2 controls *SLC7A11* transcription through nuclear accumulation, heterodimerization with transcriptional partners, and binding to antioxidant response elements within promoter regions [89]. NRF2 overexpression has been shown to increase SLC7A11/xCT protein levels, thereby enhancing GSH biosynthesis and conferring protection against oxidative injury (Figure 10) [90]. In cells stimulated with FPR2 agonists, elevated nuclear NRF2 levels were observed, suggesting that FPR2 modulates SLC7A11/xCT expression also at the transcriptional level [85].

### 3.4. FPR2 Regulates SLC1A5/ASCT2

Glutamine serves as a critical precursor for the biosynthesis of proteins, nucleotides, and amino sugars, supports cellular detoxification pathways, and contributes carbon to the mitochondrial TCA cycle for energy production [91]. Consequently, intracellular and extracellular glutamine levels are tightly regulated by a broad array of amino acid transporters. Among these, SLC1A5/ASCT2 is frequently upregulated in diverse human cancers, where it contributes to enhanced proliferative capacity [92]. Its activity is largely regulated by glutamine availability. Notably, c-Myc overexpression in cancer cells drives glutamine dependence and induces formation of a full-length farnesoid X receptor (FXR)–retinoid X receptor alpha (RXRα) heterodimer. This complex binds to the inverted repeat 1 (IR-1) element within the *SLC1A5/ASCT2* promoter, thereby promoting transcription and supporting tumor cell survival and growth [93]. c-Myc also directly enhances *SLC1A5/ASCT2* expression, and the two factors show correlated expression patterns.

In addition to transcriptional control, post-transcriptional and post-translational mechanisms contribute to SLC1A5/ASCT2 regulation. In silico analyses identify miR 137 as a key regulator targeting both SLC1A5/ASCT2 and multiple components of glutamine metabolism. Moreover, the ER-associated E3 ubiquitin ligase Ring Finger Protein 5 (RNF5) binds to SLC1A5/ASCT2 and promotes its ubiquitination and subsequent degradation, providing an additional layer of control over transporter abundance [94].

**Figure 11:**
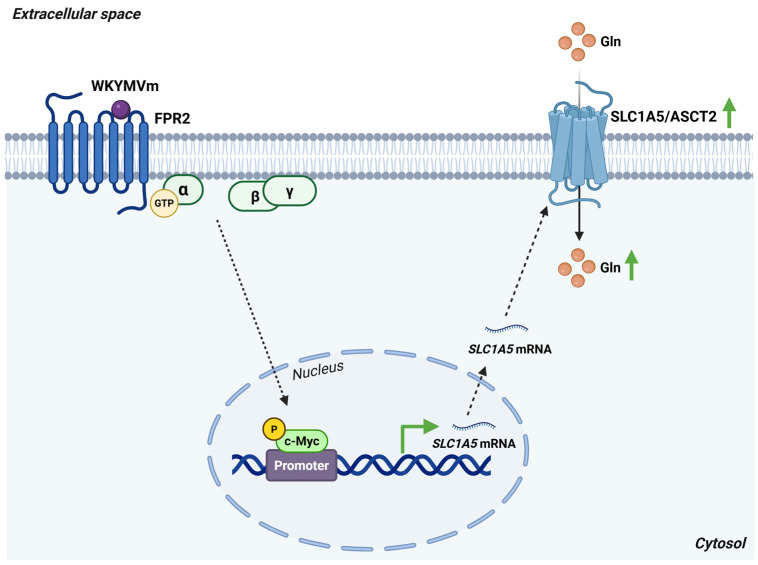
FPR2 enhances SLC1A5/ASCT2 expression and glutamine uptake through c-Myc activation. Stimulation of FPR2 by WKYMVm increases c-Myc phosphorylation and boosts its transcriptional activity, leading to elevated SLC1A5 gene expression. The resulting upregulation of SLC1A5/ASCT2 at the plasma membrane promotes glutamine uptake, thereby supporting cellular metabolic reprogramming. *Abbreviations*: ASCT2, alanine-serine-cysteine transporter 2; c-Myc, cellular myelocytomatosis oncogene; FPR2, formyl peptide receptor 2; Gln, glutamine; SLC1A5, solute carrier family 1 member 5. Created in BioRender. Cattaneo, F. (2026) License https://BioRender.com/6n1kyr8.

In FPR2-stimulated cells, a time-dependent increase in SLC1A5/ASCT2 expression has been reported [95]. Notably, FPR2 is also detected within nuclear fractions of several cell types, where its activation attenuates G_i_-dependent coupling while promoting the activation of ERK signaling, c Jun, and c-Myc [69]. Growth promoting signals drive phosphorylation of c-Myc at Ser62, a modification known to stabilize the protein and enhance its transcriptional activity [96]. Consistent with this mechanism, FPR2 stimulation induces a time-dependent accumulation of Ser62-phosphorylated c-Myc [69] (Figure 11).

Together, these findings indicate that FPR2-mediated biological responses are initiated at the plasma membrane, but may also arise from coordinated signaling events that integrate extracellular cues with intracellular, and potentially nuclear, pathways.

### 3.5. FPR2 Regulates SLC7A5/LAT1

SLC7A5 (LAT1), SLC7A8 (LAT2), SLC43A1 (LAT3), and SLC43A2 (LAT4) constitute the L-type amino acid transporter (LAT) family, which mediate the sodium-independent uptake of branched chain and aromatic amino acids [12]. LAT1 and LAT2 transport leucine, isoleucine, valine, phenylalanine, methionine, tyrosine, histidine, and tryptophan, whereas LAT3 and LAT4 primarily facilitate the uptake of leucine, isoleucine, valine, phenylalanine, and methionine [97].

In two cancer cell lines, stimulation of FPR2 induces the upregulation of SLC7A5/LAT1, thereby enhancing the cellular uptake of leucine and other essential amino acids [98]. This increase in LAT1 expression is modulated by low, transient levels of ROS generated by FPR2-dependent NADPH oxidase activity, indicating a redox-sensitive mechanism of regulation [98]. Structural analyses reveal that LAT1 contains 12 transmembrane domains with 12 conserved cysteine residues susceptible to redox modulation [99]. Among these, Cys88 and Cys439 are directly involved in transporter function, whereas Cys210 forms a critical disulfide bond with Cys164 of the heavy-chain 4F2hc, enabling the proper assembly and surface expression of the LAT1/4F2hc heterodimer [100]. Thus, the mild oxidative environment produced by FPR2-mediated NOX activation likely stabilizes LAT1 conformation and supports its function at the plasma membrane. Notably, FPR2 activation by distinct agonists also promotes a time-dependent increase in 4F2hc expression in multiple tumor cell lines [98]. These findings indicate that FPR2-driven signaling pathways coordinately regulate both components of the 4F2hc/LAT1 heterodimer, thereby enhancing amino acid transport capacity in cancer cells.

SLC7A5/LAT1-mediated uptake of leucine and other AAs activates several downstream signaling cascades, most notably the mammalian target of rapamycin (mTOR) pathway [12]. mTOR shows both serine/threonine and tyrosine kinase activity and functions through the two multiprotein complexes mTORC1 and mTORC2. In response to nutrient availability, growth factors, and other stimuli, mTORC1 phosphorylates key regulators of protein synthesis, including S6 kinase 1 (S6K1) and the eukaryotic translation initiation factor 4E-binding protein 1 (4E BP1) [101]. S6K1 undergoes hierarchical phosphorylation at multiple threonine and serine residues, enabling the subsequent phosphorylation of ribosomal protein S6 on the 40S subunit, a critical step in promoting protein synthesis, cell growth, and survival. In parallel, mTORC1 regulates cap-dependent translation through control of the 4E BP1–eIF4E interaction [102]. Under basal conditions, 4E BP1 binds to and sequesters eIF4E, preventing the assembly of the eIF4F translation initiation complex. Hyperphosphorylation of 4E BP1 by mTORC1 markedly reduces its affinity for eIF4E, thereby allowing for eIF4F complex formation and enhancing global translation rates.

FPR2 activation induces phosphorylation of both S6K1 and 4E BP1 [98]. Accordingly, the FPR2-dependent redox-regulated upregulation of SLC7A5/LAT1 enhances leucine uptake, which in turn activates mTORC1 and its downstream effectors S6K1 and 4E BP1.

**Figure 12:**
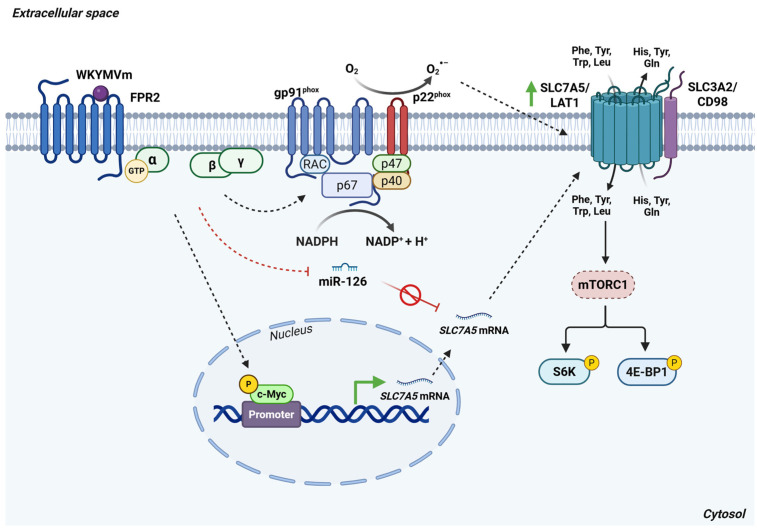
FPR2 signaling controls SLC7A5/LAT1-mediated amino acid uptake and mTORC1 activation through a NOX-dependent pathway. Stimulation of FPR2 by WKYMVm enhances c-Myc transcriptional activity, suppresses miR-126 expression and drives NADPH oxidase (NOX) activation. NOX is a multisubunit enzyme composed by gp91phox, p22phox, p67phox (p67), p47phox (p47), p40phox (p40) and RAC-GTPase (RAC). Once activated, NOX catalyzes the production of O_2_^•−^ by the one reduction of O_2_ using NADPH as an electron donor. FPR2-NOX-dependent upregulation of SLC7A5/LAT1 promotes the accumulation of SLC3A2/CD98 at the plasma membrane, facilitating the assembly of the functional LAT1/CD98 heterodimer. Together, these events increase intracellular levels of essential amino acids, such as leucine, thereby activating mTORC1 and elevating phosphorylation of its downstream effectors, 4E-BP1 and S6K. *Abbreviations*: 4E-BP1, eukaryotic translation initiation factor 4E-binding protein 1; FPR2, formyl peptide receptor 2; Gln, glutamine; His, histidine; LAT1, L-type amino acid transporter 1; Leu, leucine; mTORC1, mechanistic target of rapamycin complex 1; Phe, phenylalanine; RAC, Ras-related C3 botulinum toxin substrate 1; S6K, ribosomal protein S6 kinase; SLC3A2, solute carrier family 3 member 2; SLC7A5, solute carrier family 7 member 5; Trp, tryptophan; Tyr, tyrosine. Created in BioRender. Cattaneo, F. (2026) License https://BioRender.com/6n1kyr8.

In cancer cells, Myc is a well established transcriptional activator of *SLC7A5/LAT1* [103]. Consistently, FPR2 stimulation leads to increased c-Myc expression, suggesting that FPR2 regulates LAT1 expression at the transcriptional level [98]. SLC7A5/LAT1 expression is also controlled post-transcriptionally by miR 126, whose reduced expression contributes to elevated LAT1 levels and thereby supports the enhanced uptake of the essential amino acids required for rapid tumor growth [104]. A decrease in miR 126 levels has likewise been observed in FPR2-stimulated cancer cells [98], indicating that FPR2 influences SLC7A5/LAT1 regulation through both transcriptional and post-transcriptional mechanisms (Figure 12).

### 3.6. G Protein-Coupled Receptor 37 Like 1 Regulates GLT-1

G protein-coupled receptor 37 like 1 (GPR37L1) is an orphan GPCR expressed exclusively in glial cell populations, including satellite glial cells and astrocytes [105]. GPR37L1 has been proposed as a functional receptor for maresin 1 (MaR1), a specialized pro-resolving mediator (SPM) with potent analgesic properties in inflammatory and neuropathic pain. Binding of MaR1 to GPR37L1 elicits neuroprotective and glioprotective responses [106] and modulates potassium channel activity, thereby enhancing extracellular K^+^ buffering capacity in glial cells [107].

Glutamate transporter 1 (GLT 1), the predominant glutamate transporter in the central nervous system, plays an essential role in maintaining synaptic glutamate homeostasis. Impairments in GLT 1-mediated glutamate clearance occur prior to the formation of amyloid plaques and the onset of neuronal dysfunction, contributing to pathogenic alterations in glutamatergic signaling associated with Alzheimer’s disease [108,109]. In addition, GLT 1 serves a critical protective function against astrogliosis, and dysregulated glutamatergic transmission driven by GLT 1 dysfunction is recognized as a key contributor to chronic pain development.

**Figure 13:**
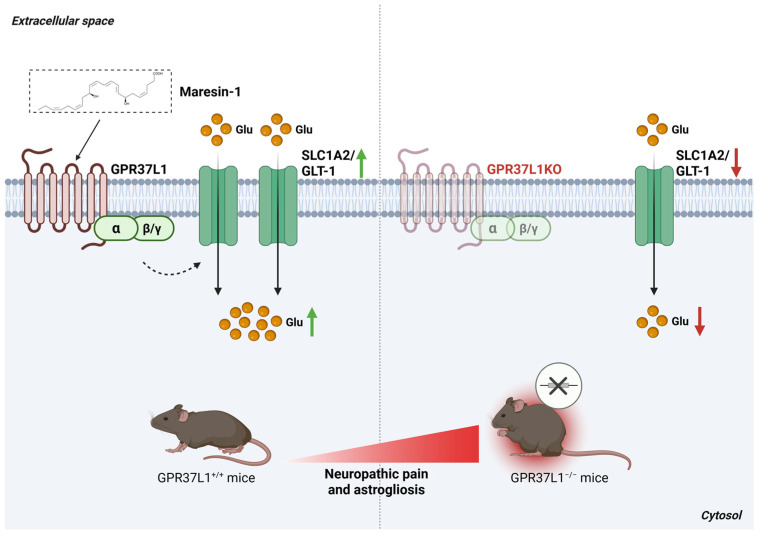
GPR37L1 regulates neuropathic pain by modulating SLC1A2/GLT-1 function in murine astrocytes. Left panel: In GPR37L1^+^/^+^ mice, activation of GPR37L1 by the pro-resolving lipid mediator maresin 1 enhances SLC1A2/GLT-1 activity in spinal cord astrocytes, increasing glutamate uptake and preventing the development of neuropathic pain. Right panel: In GPR37L1^−^/^−^ mice, loss of GPR37L1 markedly exacerbates neuropathic pain and promotes astrogliosis following nerve injury. *Abbreviations*: GLT-1, glutamate transporter 1; GPR37L1, G protein-coupled receptor 37-like 1; Glu, glutamate; SLC1A2, solute carrier family 1 member 2. Created in BioRender. Cattaneo, F. (2026) License https://BioRender.com/6n1kyr8.

Knockdown of *GPR37L1* leads to enhanced excitatory synaptic transmission and promotes the development of pain hypersensitivity, whereas *GPR37L1* overexpression is sufficient to reverse neuropathic pain [110]. Mechanistic studies indicate that both excitatory synaptic activity and neuropathic pain are regulated through the functional interaction between GPR37L1 and the glutamate transporter GLT 1. Notably, both proteins are selectively expressed in astrocytes and are downregulated following nerve injury. Restoring either GLT 1 or GPR37L1 expression prevents the onset of neuropathic pain, underscoring their cooperative role in maintaining glutamatergic homeostasis. Similarly, spinal delivery of MaR1 promotes GPR37L1 activation and facilitates GPR37L1/GLT 1 coupling, thereby preventing neuropathic pain in vivo (Figure 13) [110]. These observations highlight GPR37L1 as a promising therapeutic target and suggest that its targeted delivery, such as via lumbar puncture, may offer an effective strategy for treating chronic neuropathic pain.

## 4. Concluding Remarks

Although considerable progress has been made in defining the substrate specificity and structure–function relationships of clinically relevant solute carriers (SLCs), the mechanisms that regulate their functional expression remain comparatively underexplored. Overexpression of several SLC transporters activates oncogenic signaling pathways and supports the growth of multiple human cancers, underscoring their role in supplying essential amino acids to rapidly proliferating tumor cells. To date, most studies have focused on post-translational regulation of SLC proteins, which involves diverse molecular modifications, including phosphorylation, ubiquitination, glycosylation, and palmitoylation, that can be reversible depending on the stimulus and the desired cellular response [26]. These regulatory events frequently arise downstream of receptor-dependent signaling cascades. Among the known mechanisms, protein kinase C (PKC)-mediated pathways are the most extensively investigated and are recognized to initiate additional post-translational modifications that modulate SLC activity, trafficking, or stability [111]. Despite the fundamental role of signaling pathways in maintaining cellular homeostasis, their precise impact on transporter regulation remains insufficiently understood, highlighting an important gap in our knowledge of SLC biology.

**Figure 14:**
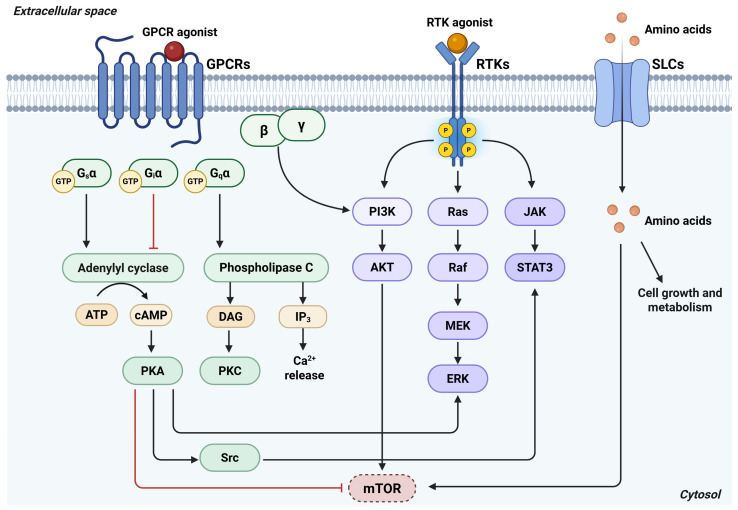
GPCRs, RTKs, and SLC transporters that engage common intracellular signaling pathways. Upon ligand binding, GPCRs activate heterotrimeric G proteins and their downstream effectors. Gα_s_ stimulates adenylyl cyclase, whereas Gα_i_ inhibits its activity. Adenylyl cyclase catalyzes cAMP production, leading to PKA activation, which, in turn, modulates multiple downstream pathways, including mTOR, Src, and MAPK signaling pathways. Gα_q_ activates PLC, generating IP_3_ and DAG, resulting in intracellular Ca^2+^ release and PKC activation. In parallel, the Gβγ dimer promotes the PI3K/Akt pathway. Upon stimulation, RTKs undergo autophosphorylation and initiate distinct signaling cascades, including the PI3K/Akt/mTOR, MAPK, and JAK/STAT pathways. SLC transporters modulate amino acid availability and are functionally integrated into this signaling network, thereby linking nutrient uptake to intracellular signaling. Overall, amino acid transport supports mTOR activation and promotes cell growth and metabolic rewiring. *Abbreviations*: cAMP, cyclic adenosine monophosphate; DAG, diacylglycerol; ERK, extracellular signal-regulated kinase; GPCR, G protein-coupled receptor; IP_3_, inositol 1,4,5-trisphosphate; JAK, Janus kinase; MEK, mitogen-activated protein kinase kinase; mTOR, mechanistic target of rapamycin; PI3K, phosphatidylinositol 3-kinase; PKA, protein kinase A; PKC, protein kinase C; RTK, receptor tyrosine kinase; SLC, solute carrier; STAT3, signal transducer and activator of transcription 3. Created in BioRender. Cattaneo, F. (2026) License https://BioRender.com/6n1kyr8.

The observation that most proteins operate within highly interconnected networks rather than as isolated entities presents a major challenge in cancer research. Extensive crosstalk between GPCR and RTKs has been shown to activate oncogenic signaling cascades, including the Ras–MAPK, PI3K–AKT, and STAT pathways. Conversely, aberrantly activated RTKs in cancer can stimulate GPCR signaling through direct interactions with heterotrimeric G proteins. While RTKs and GPCRs promote tumor progression primarily through downstream kinase activation and transcriptional reprogramming, SLCs function mainly as transporters and do not directly initiate oncogenic pathways. Nevertheless, physical and functional interactions between RTKs, GPCRs, and SLCs, as well as signaling cascades initiated by RTKs or GPCRs and modulated by SLC activity, collectively contribute to tumor cell survival and metabolic adaptation (Figure 14).

Additional layers of crosstalk arise from shared substrates. SLC expression determines the extracellular and intracellular availability of ligands for multiple GPCRs, thereby influencing receptor activation and downstream responses. Moreover, the identification of small molecules capable of targeting both GPCRs and SLCs highlights new opportunities for therapeutic intervention. The observation that RTKs and GPCRs regulate SLC activity through distinct molecular mechanisms adds further complexity to this regulatory network and offers valuable insights for the development and evaluation of targeted anticancer strategies.

## Data Availability

No new data were created or analyzed in this study. Data sharing is not applicable to this article.
